# Twitter-based measures of neighborhood sentiment as predictors of residential population health

**DOI:** 10.1371/journal.pone.0219550

**Published:** 2019-07-11

**Authors:** Joseph Gibbons, Robert Malouf, Brian Spitzberg, Lourdes Martinez, Bruce Appleyard, Caroline Thompson, Atsushi Nara, Ming-Hsiang Tsou

**Affiliations:** 1 Department of Sociology, San Diego State University, San Diego, California, United States of America; 2 Department of Linguistics and Asian/Middle Eastern Languages, San Diego State University, San Diego, California, United States of America; 3 School of Communication, San Diego State University, San Diego, California, United States of America; 4 School of Public Affairs and Fine Arts, San Diego State University, San Diego, California, United States of America; 5 School of Public Health, San Diego State University, San Diego, California, United States of America; 6 Department of Geography, San Diego State University, San Diego, California, United States of America; University of Vermont, UNITED STATES

## Abstract

Several studies have recently applied sentiment-based lexicons to Twitter to gauge local sentiment to understand health behaviors and outcomes for local areas. While this research has demonstrated the vast potential of this approach, lingering questions remain regarding the validity of Twitter mining and surveillance in local health research. First, how well does this approach predict health outcomes at very local scales, such as neighborhoods? Second, how robust are the findings garnered from sentiment signals when accounting for spatial effects? To evaluate these questions, we link 2,076,025 tweets from 66,219 distinct users in the city of San Diego over the period of 2014-12-06 to 2017-05-24 to the 500 Cities Project data and 2010–2014 American Community Survey data. We determine how well sentiment predicts self-rated mental health, sleep quality, and heart disease at a census tract level, controlling for neighborhood characteristics and spatial autocorrelation. We find that sentiment is related to some outcomes on its own, but these relationships are not present when controlling for other neighborhood factors. Evaluating our encoding strategy more closely, we discuss the limitations of existing measures of neighborhood sentiment, calling for more attention to how race/ethnicity and socio-economic status play into inferences drawn from such measures.

## Introduction

Social media such as Twitter have introduced new methodologies for measuring health behaviors and outcomes. Collectively, social media represent a relatively real-time large-scale snapshot of the messages, meanings and moods of a population. Every tweet is a signal of the sender’s state of mind and state of being at that moment. Every tweet is also an attempt at influence on the receiver’s state of mind and state of being[1]. To the extent that such communication processes succeed in influencing others, then not only do social media signal a population’s experiential state, but they also are a mechanism by which such states are socially constructed [2].

Evidence abounds that sentiment expressed in social media both signal and construct important social dynamics in society. Expressed sentiments influence a host of individual and population-level health outcomes [3,4]. These effects can include people using social media to discuss their current health as well as those expressing attitudes, which in turn, can affect the health of others. Examples of sentiment’s role on health are varied, affecting areas including food consumption [5,6], physical activity [5], drug and alcohol use [4,7,8], sleep disorders [8,9], depression [10], suicidality [11–13], heart disease [10,14], and overall mortality [10]. There are developing theories of social construction [15] and contagion [3] that implicate language itself in both reflecting and influencing such health outcomes. Sentiments derived from social media thus present great potential in the study of health.

There is a growing interest in leveraging sentiment data to measure the overall well-being of places [16]. Past research has shown that the overall mood of neighborhoods can affect health. For example, high stress neighborhoods are related to several health issues, ranging from poor sleep to coronary problems[17–24]. Based on this connection, several scholars have sought to leverage sentiment data from social media to gather the overall ‘mood’ of a neighborhood as a way to predict health outcomes, including heart disease [5,6,14]. These developments suggest exciting new means to determine the overall health in communities without having to rely on costly surveys and other obtrusive methods. However, new questions of validity arise with such sentiment measures. For example, are sentiments signaling health, or some intermediary factors that are correlated to both sentiment and health?

In establishing the usefulness of sentiment inferred from social media to determine health outcomes, there are considerations to be raised. First, how useful is aggregated emotional sentiment derived from social media? One of the key advantages of social media data are their individualized character, allowing for fine grained study of sentiment. Much of the existing publicly available health data are reported at an aggregate level, including census tracts, zip codes, and counties [16]. As such, the usefulness of sentiment is determined in part by how it can predict the aggregate well-being of a population and place. Several studies have already identified links between Twitter sentiment and health outcomes at a county level, including physical activity, obesity, diabetes, heart disease, and mortality [4,6,14,25]. However, linking Twitter health outcomes with datasets at smaller scales like census tracts, to our knowledge, has not yet been done [26–28].

Second, health outcomes are known to vary spatially, clustering more in some areas over others [29–31]. Some of this concentration is likely due to local forces such as concentrated socioeconomic disadvantage. However, certain forms of poor health, including stress, can be predicted by its neighboring presence, spilling over into a given area [32]. There also may be unmodeled spatial effects that affect health, including sentiment. It becomes important therefore to determine whether sentiment has an independent relationship to health outcomes independent of these other neighborhood effects.

This study evaluates the singular impact of neighborhood sentiment as measured by social media by comparing the relation of an established method of identifying sentiment to neighborhood health outcomes, including self-rated mental health, sleep quality, and heart disease as exemplars. There are relatively well-established relationships between sentiment and sleep disorders or deprivation [26], and significant inroads are progressing in sleep disorder surveillance of social media language [9]. Likewise, mental health indicators [27] such as depression can both be located linguistically in sentiment from social media [28,33–35] and associated with social media use [36]. Finally, mining of sentiments expressed in social media has demonstrated robust relationships with heart disease and cardiac-related illness [4,6,10,14]. Thus, these three measures were chosen first for their interrelationships to social media communication processes, and second for their diversity in effects: self-rated mental health being related to well-being, poor sleep as a social behavior, and heart disease a physical health outcome. These variables allow a valuable window for examining whether and how local sentiment relates to local health. In turn, this approach can establish how well sentiment predicts health outcomes when controlling for relevant neighborhood factors.

## Data and methods

### Study location

For this study, we focus on the city of San Diego, CA. While San Diego has a large population, 1,307,402 based on the 2010 Census, its built environment varies from a dense urban core to lower density suburban stretches. There is also considerable demographic variation in the city. Based on our analysis of census tract-level 2010–2014 American Community Survey data for San Diego, we found that while the Southeastern sections of the city are mostly non-White and low-income, the Northwestern sections of the city are Whiter and more affluent. This diversity in built environment and demographic environment makes San Diego an ideal site for study. The unit of analysis for this study was the census tract. Tracts were chosen because the health outcome data were derived from this local scale, as described below. Tracts are also useful as they are a common proxy of neighborhoods in city research [37], allowing greater generalizability of our findings. One consideration with San Diego is that there is a section of the city that is not connected to the rest. This ‘island’ is problematic for the spatial weighting used in this analysis discussed below, which requires that all neighborhoods share borders. As such, we omitted southern sections of the city from our final analysis. In addition, tracts for which fewer than 1,000 tweets were collected have been excluded. These omitted tracts accounted for only 7.77 percent of all the tracts in the city. Our final dataset includes a total count of 281 census tracts.

### Measuring neighborhood sentiment

**Fig 1:**
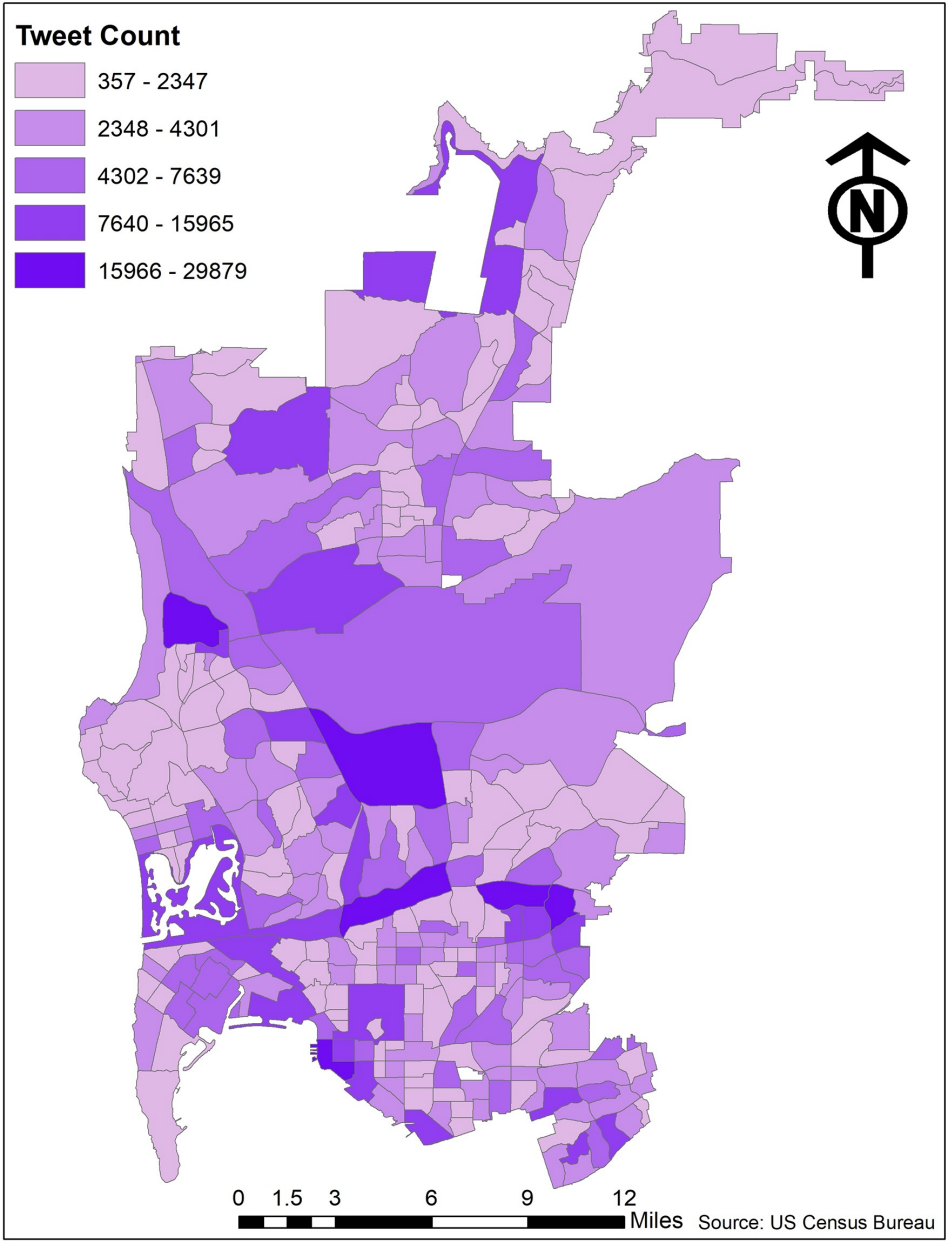
Number of tweets collected by census tract.

To measure the emotional sentiment of neighborhoods, we leveraged the content of Twitter data. Twitter is a short-form blogging system, which had until recently been limited to 140 characters a post. Geo-referenced tweets for this study were collected using the web-based application Geoviewer [38]. All data use was consistent with user expectations as per Twitter Terms of Service. Several steps were made to prepare these data for analysis. Tweets that could be located with a census tract in the parts of San Diego studied were filtered by matching the source against a whitelist of interactive Twitter applications. The accepted clean source strings were: Fenix for Android, Flamingo for Android, Tweetbot for Mac, Tweetbot for iOS, Tweetings for iPad, Tweetings for iPhone, Twitter for Android, Twitter for iPhone, Twitter for Android, Twitter for Android Tablets, Twitter for BlackBerry, Twitter for BlackBerry, Twitter for Windows, Twitter for Windows Phone, Twitter for iPad, Twitter for iPhone. This led to the exclusion of tweets from automated services that post job ads, traffic updates, earthquake reports, and such. It also excluded automated cross-posts from other social media platforms such as Instagram and FourSquare, as well as duplicate tweets. As these tweets were not collected randomly, there is the potential for sampling bias in our results. The final database included 2,076,025 tweets from 66,219 distinct users over the period 2014-12-06 to 2017-05-24. Fig 1 shows the number of tweets collected in each of the 281 census tracts in central San Diego.

To measure the overall sentiment of aggregated tweets, we applied the ‘Hedonometer’ developed by Dodds and colleagues [39,40]. This method was chosen in part because it had been used in previous tract-level studies on health [5]. This approach uses a large lexicon of more than 10,000 frequently occurring words annotated for sentiment by human raters. Each word was rated independently on a “happiness” scale of 1 to 9 (ranging from least to most positive) by 50 users on Amazon’s Mechanical Turk platform, yielding a human-derived average happiness rating *h*_avg_. To increase the metric’s robustness against random variation between raters and texts, Dodds et al. ignore words with an *h*_avg_ rating between 4 and 6 (i.e., within ±1 point of the hypothetical neutral value 5). This leaves a vocabulary of 3,731 coded words, which Dodds et al. released as the *labMT 1*.*0* data set.

Given the average happiness ratings of individual words, the average happiness of a text is simply the weighted average of the happiness ratings of the constituent words. More specifically, the average happiness of a text *T* is

$$h_{avg}(T)=\frac{\sum_{i=1}^{N}h_{avg}(w_{i})f(w_{i})}{\sum_{i=1}^{N}f(w_{i})}$$

where *h*_avg_(*w*_*i*_) is the happiness rating for the *i*th word in *labMT* and *f*(*w*_*i*_) is the frequency of that word in *T*.

It should be noted that the Hedonometer was initially designed to measure happiness at a larger scale than that used in this study, such as states [39]. Nonetheless, the highly local focus used in this study allows us to asses local issues in the derivation of *h*_avg_(*T*) scores that may not be identified otherwise.

While our central interest is in annotated lexicons, there is also a question to be raised as for how these lexicons differ from those derived from supervised machine learning. Sentiment models derived from supervised machine learning are learned from a representative distribution of words occurring and may not be subject to the annotator biases found with the *h*_avg_. To evaluate the applicability of our findings with the *h*_avg_ to supervised lexicon methods, we utilize a supervised machine learning system, the VADER (Valence Aware Dictionary for sEntiment Reasoning) in supplemental analysis[41].

### Measuring health outcomes

Our three outcome variables include *poor self-rated mental health*, the percentage of respondents 18 or over “who report 14 or more days during the past 30 days during which their mental health was not good;” *poor sleep*, the percent of respondents 18 and over who sleep less than 7 hours during a 24 hour period; and *heart disease*, the percent of respondents 18 and over who “report ever having been told by a doctor, nurse, or other health professional that they had angina or coronary heart disease.” These measures were derived from the 500 Cities Project, an initiative on the part of the Center for Disease Control and Prevention (CDC) to provide local level estimates of health risks, health outcomes, and healthy behaviors based on the 2014 wave of the Behavioral Risk Factor Surveillance System (BRFSS), a nationally representative household telephone survey administered by the CDC. Tract and city estimates from the BRFSS were derived through multilevel strategy linking geocoded county-level BRFSS data to block-level demographic data from the 2010 Census to predict the characteristics of health by location [42].

To validate this method of data creation, the CDC created county-level estimates out of their local area estimations and compared them to the raw BRFSS estimates for counties in Missouri [43] and Massachusetts [44]. They found these measures closely paralleled one another. Thus far, tract estimates have only been generated for the 2014 BRFSS data.

### Demographic measures

Demographic measures were obtained from the 2010–2014 American Community Survey. Given the level of collinearity that can exist between aggregated measures, care was taken to identify variables with the least collinearity. First, based on previous research on neighborhood context and health outcomes [45], we derived a composite measure of *socio-economic status* derived from principal component analysis of percent of tract living in poverty (loading -0.77), percent with a professional degree (loading 0.91), percent with a bachelor’s degree or greater (loading 0.90), median household income (loading 0.89), median rent (loading 0.80), and median household value (loading 0.84). This component accounts for 73.74% of the common variance in the variables. Tract-level scores were derived through the regression method [46]. In addition, we accounted for the percent of the population with some form of *insurance*, the percent *female*, the percent aged *50 and over*, and percent *nonwhite*.

#### Measuring the built environment

Most travel behavior and built environment research currently relies on the “D-variables,” first developed by Cervero and Kockelman, who originally coined the first three variable names—density, diversity, and design [47]. Based on this approach, we used measures of *regional accessibility* through a) the number of jobs accessible within a 45-minute trip by transit, and b) the number of jobs within a 45-minute trip by auto. Regional accessibility is one of the strongest predictors of lowering auto use. For walkability and bike-ability, we used intersection density, which is often used as a reliable proxy [48–50].

### Analytical approach

We used multivariate generalized linear models to identify how neighborhood attributes like aggregated sentiment affect population-level screening behaviors. To manage the spatial autocorrelation in our results, this study makes use of Exploratory Spatial Dependence Analysis (ESDA), specifically Local Indicators of Spatial Autocorrelation (LISA), to determine the presence of spatial autocorrelation and Spatial Regression to model for any local interference in the results [51]. There are two estimation strategies to manage spatial dependence in regression models: the first seeks to account for spatial lag by including a lag term, the standardized levels of the dependent variable in adjacent areas, ρ, into the model as a predictor; the second strategy incorporates a spatial error term, λ, to filter out the effects of autocorrelation from the model[52–54]. Through a series of Lagrange multiplier tests suggested by Baltagi et al.[55], we determined that spatial dependence was best accounted for by both spatial lag and spatial error. We accounted for both forms with Spatial Autoregressive Model with Autoregressive Disturbances (SARAR) that includes terms for spatial lag and error as outlined by Kelejian and Prucha [56]. The model takes on the form:

$$y_{n}=X_{n}\beta_{n}+\lambda_{n\;}W_{n}y_{n}+u_{n}$$

$$=Z_{n}\delta_{n}+u_{n}$$

and

$$u_{n}=\rho_{n}M_{n}u_{n}+u_{n}$$

with Z_*n*_ = [X_*n*_, W_*n*_y] and δn = [β′n,λn]'. Here y_*n*_ denotes the *n*× 1 vector of observations of the dependent variable, X_*n*_ denotes the *n* × *k* matrix of non-stochastic (exogenous) regressors, W_*n*_ and M_*n*_ are *n* × *n* non-stochastic matrices, u_*n*_ denotes the *n* × 1 vector of regression disturbances, ε_*n*_ is an *n* × 1 vector of innovations, λ_*n*_ and ρ_*n*_ are unknown scalar parameters, and β_*n*_ is a *k* × 1 vector of unknown parameters. The matrices W_*n*_ and M_*n*_ are typically referred to as spatial weights matrices, and λ_*n*_ and ρ_*n*_ are typically called spatial autoregressive parameters. The analysis allows for W_*n*_ = M_*n*_, which will frequently be the case in applications. The vectors ȳ_*n*_ = W_*n*_y_*n*_ and ū_*n*_ = M_*n*_u_*n*_ are typically referred to as spatial lags of y_*n*_ and u_*n*_, respectively. We note that all quantities can depend on the sample size and so some of the exogenous regressors may be spatial lags of exogenous variables. Thus, the model is relatively general in that it allows for spatial spillovers in the endogenous variables, exogenous variables and disturbances.

Analyzing aggregate measures such as these limits the ability to make claims about individual level outcomes because of the potential for ecological fallacy and the modifiable areal unit problem [57,58]. Arguments and assumptions therefore need to be reserved to group-level effects.

## Results

**Fig 2:**
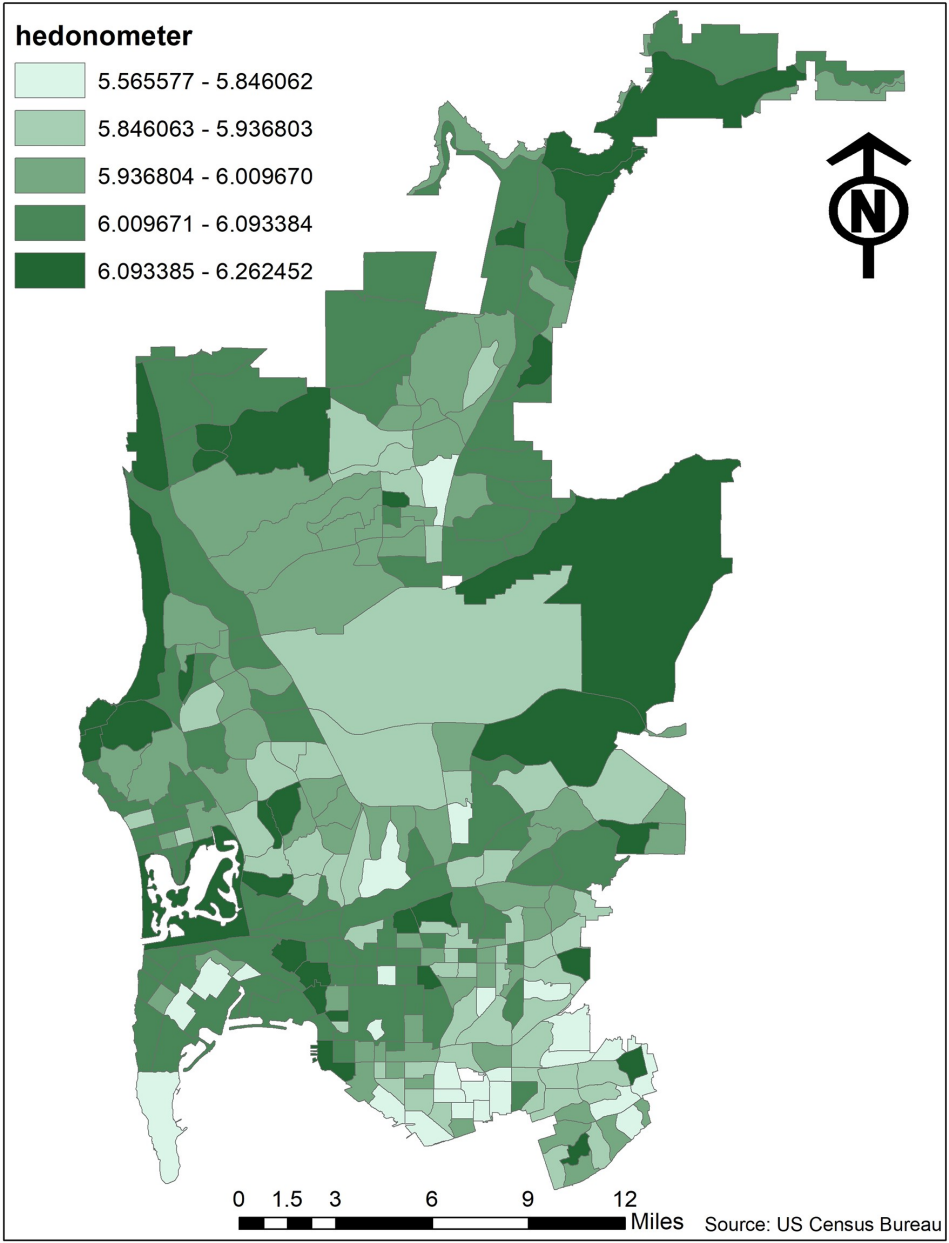
h_avg_ by census tract.

The descriptive findings are reported in Table 1. First, the hedonometer grand mean score for a census tract in the measured sections of San Diego (*h*_avg_) was 5.985. We visualize the distribution of *h*_avg_ scores by tract with Fig 2. On average 10.694 percent of the measured tracts report poor self-rated mental health, though some tracts have as much as 20.600 percent reporting poor self-rated mental health. Next, on average 33.139 percent of the measured tracts report poor sleep, with some tracts reporting as many as 44.200 percent. Last, on average 4.540 percent of tract residents report heart disease, with as many as 13.500 percent in some areas. In sum, there is a fair amount of variation in the health outcomes in the tracts across the measured sections of San Diego.

**Table 1:** Descriptive statistics.

Variable	N	Mean	St. Dev.	Min	Max
Self-Rated Mental Health	281	10.694	3.750	0.000	20.600
Poor Sleep	281	33.139	7.940	0.000	44.200
Chronic Heart Disease	281	4.540	1.829	0.000	13.500
*h*_avg_	281	5.985	0.100	5.566	6.262
VADER	281	0.000	1.000	-2.140	3.640
Insurance	281	0.848	0.123	0.000	1.000
Proportion Over 40	281	0.294	0.125	0.000	0.952
Socio-economic Status	281	0.605	2.287	-4.359	5.667
Proportion Nonwhite	281	53.585	26.557	1.942	95.472
Automobile Access	281	566,819.200	232,542.900	21,912.440	1,269,017.000
Rail Access	281	22,828.960	24,012.750	0.000	170,026.200
Intersection Density	281	273.018	169.934	2.391	1,398.743

**Fig 3:**
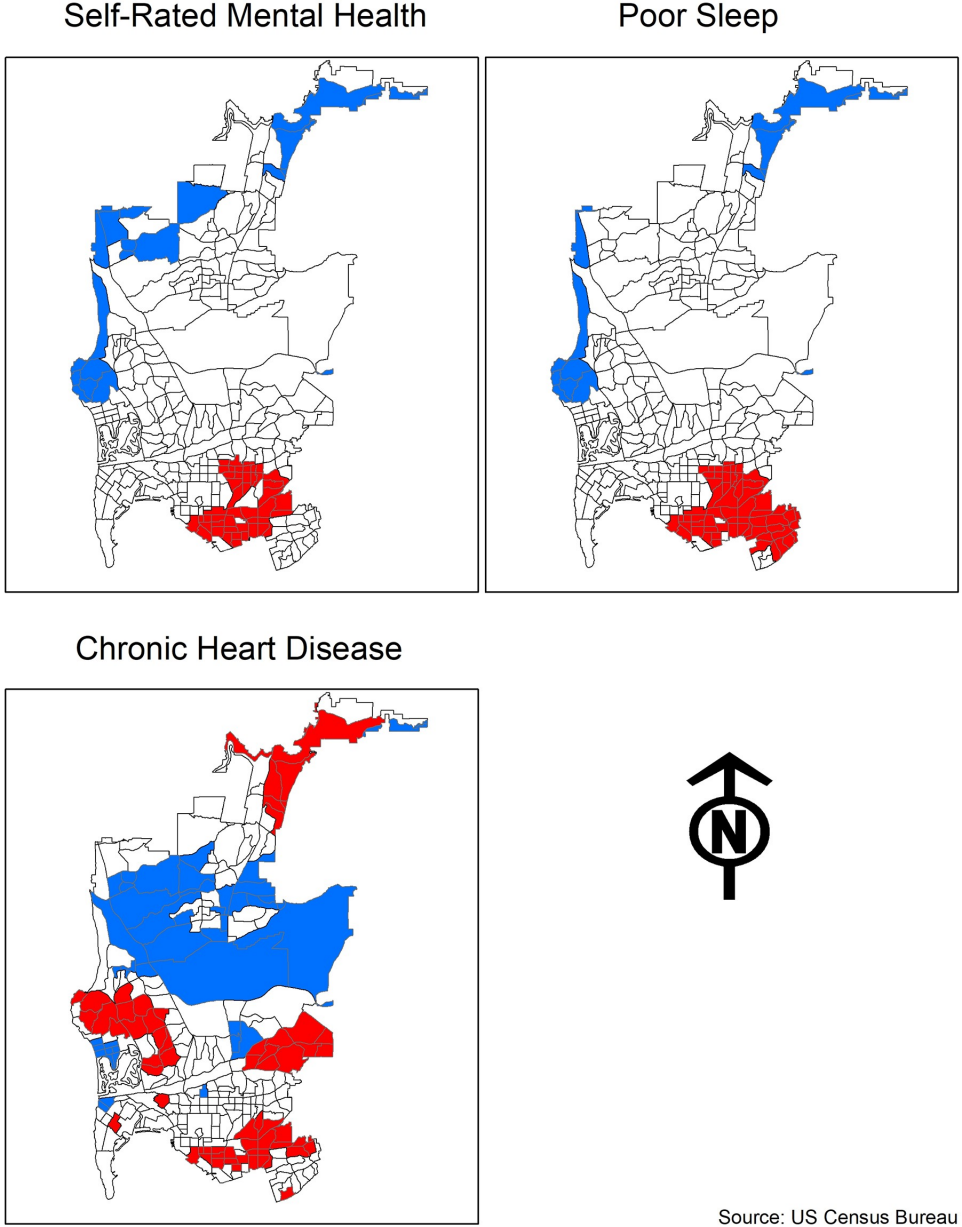
Exploratory spatial data analysis.

We utilized Exploratory Spatial Data Analysis (ESDA) to determine the underlying spatial autocorrelation in our outcomes. Across all three measures we found significant (p≤ 0.001) and moderate spatial autocorrelation with self-rated mental health (0.542), poor sleep (0.339), and heart disease (0.239). To further assess the presence of these clusters, we utilize Local Indicators of Spatial Autocorrelation (LISA), which displays the local iterations of the Moran’s I scores. Presented in Fig 3, these maps display clearly demarcated spatial clusters of significantly higher poor health (High-High) and areas that significantly lack poor health (Low-Low). To clarify, the Low-Low areas do not necessarily have high rates of good health, but they do lack unhealthy people. Self-rated mental health and poor sleep present a similar spatial pattern, with the High-High areas mainly in southeastern San Diego and the Low-Low areas mainly to the North and West of the city. Heart disease displays a different pattern, with four large High-High clusters. While one of these clusters is in southeastern San Diego, another is in the western reaches of the city, which contained the Low-Low clusters for mental health and sleep.

We report our regression results in Table 2; Models 1, 4, and 7 are OLS findings of the *h*_avg_ measure with the health outcomes. Comparisons of the *h*_avg_ coefficients across models were assessed using the technique described by Clogg, Petkova, and Haritou [59]. We find based on Models 1 and 4 that the *h*_avg_ has significant and *negative* self-rated mental health (-1.294***) and poor sleep (-.2.118***) respectively. Meanwhile, as shown in Model 7 *h*_avg_ has no significant relationship with chronic heart disease. The negative relation of happiness to these outcomes is notable, model 1 for example implies that tracts with ‘happier’ Twitter activity are reporting worse self-rated mental health. A post regression analysis reveals the residuals of the OLS were significantly (p≤0.001) spatially autocorrelated, indicating bias in our estimations not being accounted for.

**Table 2:** Multiple regression results for health outcomes—Hedonometer.

	Self-Rated Mental Health			Poor Sleep			Chronic Heart Disease		
	*OLS*	*SARAR*		*OLS*	*SARAR*		*OLS*	*SARAR*	
	(1)	(2)	(3)	(4)	(5)	(6)	(7)	(8)	(9)
*h*_avg_	-1.294***	-0.421***	0.093	-2.118***	-0.931**	-0.060	0.094	0.110	-0.025
	(0.211)	(0.155)	(0.165)	(0.458)	(0.395)	(0.446)	(0.109)	(0.100)	(0.091)
Insurance			-0.203			0.333			-0.495***
			(0.201)			(0.544)			(0.111)
Percent 50 and Over			-0.514***			-1.384***			1.321***
			(0.164)			(0.446)			(0.098)
SES			-1.215***			-0.053			-0.387**
			(0.294)			(0.759)			(0.158)
Percent Nonwhite			-0.244			-2.023***			-0.019
			(0.230)			(0.635)			(0.124)
Auto Transit Access			0.068			0.392			0.121
			(0.216)			(0.586)			(0.120)
Public Transit Access			0.110			0.562			-0.0004
			(0.219)			(0.589)			(0.120)
Intersection Density			-0.025			0.022			-0.053
			(0.202)			(0.548)			(0.112)
Constant	10.694***	2.576***	6.080***	33.139***	12.278***	19.090***	4.540***	2.366***	3.440***
	(0.210)	(0.486)	(0.678)	(0.457)	(1.941)	(2.365)	(0.109)	(0.338)	(0.320)
Observations	281	281	281	281	281	281	281	281	281
Log Likelihood		-682.847	-640.769		-939.967	-920.524		-550.137	-470.290

Note:

*p<0.1;

**p<0.05;

***p<0.01; Predictors are Standardized

Using a Lagrange multiplier test [55], we determined that the SARAR model [56] was the best estimation strategy for our models, which are reported in Models 2, 5, and 8. These Models show that the *h*_avg_ is still significant in predicting self-rated mental health and poor sleep, but the magnitude of the effects is notably smaller. The *h*_avg_ for self-rated mental health ranges from -1.294 in Model 1 to -0.421 in Model 2. These differences were statistically significant. Finally, the *h*_avg_ has no significance in these models when other neighborhood controls are added, as reported in Models 3, 6, and 9. The most consistent effect explaining the effects of these outcomes is age, which is significant for all the models.

To determine the applicability of our findings to lexicons derived from supervised machine learning, we conducted supplemental analyses, reconducting our models using the VADER [41] in place of the *h*_avg_. These results, reported in Table 3, are largely consistent with the models reported in Table 2, with the VADER measure significantly predicting self-rated health and sleep in base models but losing significance in full models. The similarity of the VADER results to our reported results using the *h*_avg_ suggest the bias we identified with annotated lexicons is also applicable to at least some of the existing supervised machine learning methods.

**Table 3:** Multiple regression results for health outcomes—VADER.

	Self-Rated Mental Health			Poor Sleep			Chronic Heart Disease		
	*OLS*	*SARAR*		*OLS*	*SARAR*		*OLS*	*SARAR*	
	(1)	(2)	(3)	(4)	(5)	(6)	(7)	(8)	(9)
VADER	-0.892***	-0.285*	0.102	-1.411***	-0.558	0.158	0.137	0.147	0.029
	(0.224)	(0.158)	(0.157)	(0.479)	(0.404)	(0.425)	(0.112)	(0.103)	(0.087)
Insurance			-0.205			0.341			-0.492***
			(0.201)			(0.543)			(0.111)
Percent 50 and Over			-0.513***			-1.414***			1.313***
			(0.164)			(0.444)			(0.097)
SES			-1.206***			-0.111			-0.403***
			(0.290)			(0.746)			(0.155)
Percent Nonwhite			-0.248			-2.047***			-0.026
			(0.230)			(0.635)			(0.124)
Auto Transit Access			0.068			0.388			0.120
			(0.216)			(0.585)			(0.119)
Public Transit Access			0.109			0.528			-0.009
			(0.218)			(0.589)			(0.120)
Intersection Density			-0.023			0.013			-0.055
			(0.202)			(0.547)			(0.112)
Constant	10.694***	2.350***	6.109***	33.139***	11.428***	19.013***	4.540***	2.367***	3.438***
	(0.218)	(0.467)	(0.677)	(0.467)	(1.877)	(2.358)	(0.109)	(0.338)	(0.319)
Observations	281	281	281	281	281	281	281	281	281
Log Likelihood		-684.661	-640.718		-941.605	-920.464		-549.719	-470.272

Note:

*p<0.1;

**p<0.05;

***p<0.01; Predictors are Standardized

The above results raise a few notable points. First, we find that the baseline measure of the *h*_avg_ has an unexpectedly positive association with poorer health outcomes. Put simply, neighborhood happiness as measured by Twitter activity in a neighborhood was associated with worse rather than better health as measured by self-rated poor mental health and poor sleep, though not by heart disease, which was unrelated to Twitter happiness. Second, this association between Twitter happiness and poor health significantly weakens in magnitude when spatial autocorrelation is estimated, and the remaining association loses all significance with the introduction of the controls. On the surface, these results demonstrate the limitation in the ability of sentiment measures to predict neighborhood outcomes independent of other neighborhood factors. However, a closer evaluation of these sentiment measures reveals more about how and why they were not successful predictors.

## Context and sentiment

Lexicon-based sentiment analysis metrics like the Hedonometer suffer from a number of limitations (see Pang and Lee [60], for a survey). Many of these come down to an inability to properly take context into account. That could be the immediate linguistic context: for example, the phrase *not happy* would be assigned a moderately positive happiness score, while *not unhappy* would be judged strongly negative. The strictly additive combination of sentiment scores assumed by these methods cannot account for the semantics of natural language use.

More generally, lexicon-based methods deal poorly with polysemy, the situation in which a single word has multiple related meanings with potentially different sentiments attached. For example, the word *animals* is moderately positive, with an *h*_avg_ of 6.80, and it is in fact usually used in a positive sense:

baby **animals** and beautiful sunsets …this place is magical #newfriends #shouldhavebeenavet

It is easy, though, to find examples of the same word being used in a strongly negative sense:

I can't stand people who don't control their fucking children in public places. have them act like fucking **animals** in your home

Sentiment lexicons that are derived automatically from text typically average sentiment scores across all possible meanings of a word. It is hard to know exactly how the MTurkers who coded the labMT lexicon approached the problem, but they likely either (impressionistically) averaged across word senses or, alternatively, assigned each word an *h* score that reflects the word’s most salient sense.

These shortcomings (and others) make a system like the Hedonometer unsuitable for accurately assigning absolute happiness scores to small texts. However, they might not be a problem when the system is used to compare relative levels of happiness across large quantities of text distributed across space or time, as long as errors in sentiment are not correlated with any other dimensions of interest. For example, negative uses of *animals* may add noise to overall happiness measurements, but they do not pose a problem for trend analysis so long as the ratio of positive to negative uses of *animals* remains constant over time.

For the most part, the assumption that word sense probabilities are stationary has gone unexamined in the literature in large-scale social media analysis, though sporadic violations are occasionally noted. Dodds et al. [39] cite the example of an increase in negative sentiment on May 24, 2010. This was the date of the series finale of the TV drama *Lost*, an event that generated a lot of social media interest. The word *lost* is negative in most of its senses and has a fairly negative score (*h*_avg_ = 2.76), but on that date the neutral-to-positive ‘TV show’ sense of the word increased in relative frequency at the expense of the other senses. This shift in word-sense probabilities possibly led to a spurious spike in negative sentiment and certainly made it difficult to measure whether the end of *Lost* was actually met with a global drop in happiness.

In this analysis, we are considering variations in happiness over space rather than time, and there is good reason to suspect that word-sense probabilities are not (spatially) stationary. Like all large American cities, San Diego is both a multi-lingual and a multi-dialectal community. The tweets we collected represent usage in multiple varieties of African American English (AAE), Chicano English, and Standard American English (SAE), among other dialects. Words and word meanings vary across dialects, and the dialects in a tweeter’s linguistic repertoire depend in part on their location, class, and ethnicity.

To investigate the underlying causes that lead to variation in happiness measurements, Dodds and Danforth [40] introduced the **word shift graph**, a visualization that shows the words that contribute most to differences in happiness. A word can contribute to higher happiness in two ways: a word with a higher than average *h*_avg_ can occur more frequently than average, or a word with a lower than average *h*_avg_ can occur less frequently than average. Similarly, more frequent negative words and less frequent positive words contribute to a decrease in measured happiness. Specifically, the normalized per-word happiness shift *δh*_avg,*i*_ of a word *w*_*i*_ to the difference in happiness *δh*_avg_ between a comparison text *T*_comp_ and a reference text *T*_ref_ is:

$${\delta h}_{avg,\;i}=\frac{100}{\left|h_{avg}^{\left(comp\right)}-h_{avg}^{\left(ref\right)}\right|}[h_{avg}(w_{i})-h_{avg}^{\left(ref\right)}][p_{i}^{\left(comp\right)}-p_{i}^{\left(ref\right)}]$$

where $h_{avg}^{\left(comp\right)}$ and $h_{avg}^{\left(comp\right)}$ are the average happiness of *T*_comp_ and *T*_ref_ and $p_{i}^{\left(comp\right)}$ and $p_{i}^{\left(ref\right)}$ are the relative frequencies of *w*_*i*_ in *T*_comp_ and *T*_ref_.

**Fig 4:**
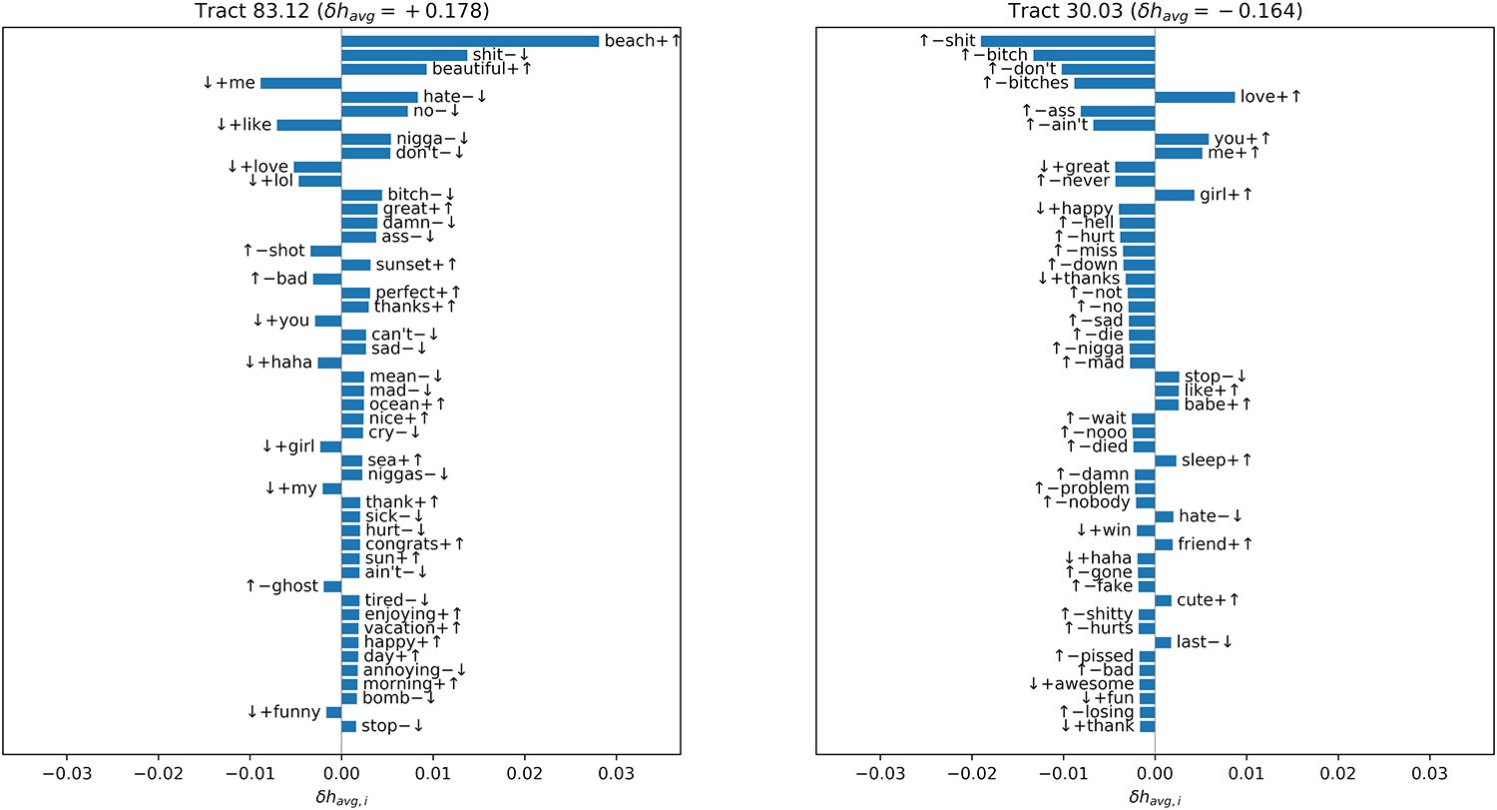
Word shift graph.

Word-shift graphs for census tract 83.12 (in La Jolla, a wealthy coastal community) and 30.03 (part of Encanto, a working class and more rural inland neighborhood) are given in Fig 4. For each word, the size of the bar indicates the magnitude of *δh*_avg,*i*_ and the direction indicates its sign. Words that are more or less frequent than average in the given tract are marked with ↑ or ↓ respectively, and words with *h*_avg_ greater or less than $h_{avg}^{\left(ref\right)}$ are marked with + or −.

In tract 83.12 we see an increased frequency of words reflecting the physical environment and the positive things people do there (*beach*, *beautiful*, *great*, *sunset*, *perfect*, *thanks*, *ocean*, *nice*, *sea*, *congrats*, *sun*, *enjoying*, *vacation*) and a reduced frequency of words denoting negative affects (*hate*, *sad*, *mad*, *cry*, *mean*, *sick*, *hurt*, *tired*, *annoying*).

The words with high *δh*_avg,*i*_ in tract 30.03 are not as straightforward to interpret. This list is not particular to tract 30.03. In fact, the 20 words most responsible for contributing to *δh*_avg_ across all census tracts, reflect many of the same terms:

shit, love, don’t, no, happy, me, nigga, lol, hate, not, like, bitch, can’t, ass, great, haha, damn, never, niggas, dont

Two words stand out immediately: *nigga* and *niggas*. The semantic and pragmatic status of these terms depends substantially on the identity of the speaker using them, their addressee, and the context of use. These terms are both rated as strongly unhappy in labMT, with *h*_avg_ of 3.32 and 2.96, and this is probably an accurate reflection of the linguistic experience of the raters. However, among speakers of AAE (and other dialects), these terms have undergone a kind of ‘semantic bleaching’ in which they have lost most of their original meaning and have come to be used in some cases, arguably, as a kind of pronoun [61–64]. Further, other research has shown a clear gendered difference in the use of these words, with men using them at a far greater rate than women [65]. The use of these terms is an indicator of the tweeter’s dialect (and, less directly, race and socio-economic status [66]), not of their level of positive emotion.

Several more of the top words (*shit*, *bitch*, *ass*, *damn*) are swear words. What counts as profanity varies across dialects. Swearing also serves many functions, and the expression of negative affect such as anger is certainly one of them [65]. Use of profanity can express solidarity and it can also serve an indexical function in the construction of a social identity [64,67,68].

In her study of profanity use among college students, Beers Fägersten [69] found that *shit* was the swearword used most often (by a wide margin) by African Americans in her sample, accounting for 44% of the total profanity use, and much more often than among White, Hispanic, or Asian-American students, for whom *fuck* was the most frequently used term. Even though *fuck* is normally seen as one of the most offensive profanities in American English [70], it received a fairly neutral rating of 4.14 in labMT, whereas *shit* was rated very negatively at 2.50. Again, this is probably an accurate representation of SAE as judged by the raters (on average), but it does not reflect usage in other dialects or contexts. In addition, Beers Fägersten [69] observed differences in the context of swearing between racial groups. The range of functions of profanity was the same across groups, but African American students were more likely than members of other ethnicities to use swearing in among friends and in humorous or emphatic way. Profanity use, as an indicator of mood, is not constant across dialects.

A third category of words on the list is made up of negation terms (*don’t*, *no*, *not*, *can’t*, *never*, *dont*). Grammatical differences between SAE and non-standard dialects may be influencing the frequencies of specific negative terms [71]. For example, forms like *She don’t look 18* (≈ SAE *She doesn’t look 18*) may account for the over-representation of *don’t* and *dont* in some tracts. Similarly, the negative items *no* and *never* in some dialects correspond to *a*/*any* and *ever* in the standard dialect: *being searched ain’t no joke ≈ being searched isn’t a joke; You ain’t never going to be happy ≈ you aren’t ever going to be happy*. *No* (*h*_avg_ = 3.48) and *never* (*h*_avg_ = 3.48) are negatively rated in labMT while *a*, *any*, and *ever* have ratings very close to 5.

One possible objection that could be raised at this point is that the hedonometer was originally intended to be applied to aggregations of Twitter users over areas much larger than a census tract. Zooming out to larger geographies, however, does not eliminate these local inconsistencies. For example, Mitchell et al. [72] compare hedonometer scores across US states. If we look at the word shift graph for Mississippi, the state with the lowest *h*_avg_ score, we see that the most single influential word is *gone*. In this context, many of the uses of *gone* are as an AAE future tense marker, similar to *gonna* or *going to* in SAE [73,74]. It should be noted that Mississippi also has the highest share of Blacks than any other state in the United States. This use of *gone* has *h*_avg_ = 3.42), but it should probably be neutral (as *gonna* and *going* are). Other top words influencing Mississippi’s low *h*_avg_ are *shit*, *ain’t*, *ass*, *damn*, *hell*, *bitch*, and *nobody*, which are discussed above. We would argue that issues raised by dialect variation are exaggerated when looking at small areas and populations, but they exist and need to be accounted for at any scale.

This evidence suggests that word-sense does vary with dialect, and therefore also with neighborhood and demographic variables class, race, age, and gender. Furthermore, non-standard dialect forms are judged systematically as less happy than standard dialect usages. This raises a challenge for interpreting our results: when happiness is measured using word ratings calibrated to an SAE norm, what may actually be measured, in part, is race and class. This calls for more sophisticated hedonometric analysis techniques that can isolate the effects of emotion from dialect variation [66,75–77]. A simple approach would be to identify and remove from the lexicon terms that have a strong association with a particular demographic group. However, the hedonometer rating for all words is affected by dialect variation and racial, ethnic, and class bias to some degree. Even usage of social media varies based upon class, with lower income populations using platforms like Twitter for different reasons than upper income populations [66]. Removing the most obviously problematic words only makes the problem more difficult to detect. An alternative strategy would be to use the frequency of these words as an indirect indicator of ‘dialect’ among these demographic groups, using Bayesian approach to sort out the potential bias of each word [73].

The unexpected negative relationship between happiness and health requires further interpretation. An analogous anomalous finding occurred in Eichstaedt et al. [14], where they found that a LIWC index of positive relationship language correlated positively to mortality. They speculated that this might be due to proportionally higher use of positive relationship language in lower-SES census tracts. Other research, however, has tended to find that indicators of happiness and satisfaction in Twitter tend to correlate in expected ways to both socio-demographics and to healthy behavior, morbidity and mortality, even when controlling for such demographics [2,4–6]. Thus, our finding that spatial autocorrelation and neighborhood controls affect the relationship between Twitter happiness and health correlates indicates the importance of controlling for such factors when investigating the relationship between sentiment expressed on social media and health.

## Conclusion

The goal of this paper was to evaluate the usefulness of Twitter-based measures of sentiment to predict health outcomes. While the sentiment identified in Twitter has been linked with county-based health outcomes, existing studies are limited in several key ways. Past studies have not examined the relationship of sentiments expressed via Twitter to health outcomes at the neighborhood level, nor have they accounted for the possible spatial autocorrelation that may impact, or explain away, this relationship. This study sought to address these limitations by leveraging Twitter data from San Diego, CA to measure emotional sentiment in neighborhoods to determine whether sentiment in a neighborhood relates to select health outcomes for that neighborhood. To measure sentiment, we drew on the hedonometer, a human coded system that rates words on a happiness scale of 1 to 9, ranging from least to most positive. We found that the average hedonometer score for census tracts (*h*_avg_) has no predictive power on measuring health outcomes when accounting for neighborhood-level effects in San Diego. Further, in post analysis discussion we note the deep bias that exists within the construction of the hedonometer estimates along the lines of race and class.

These findings do not necessarily imply that the aggregate emotional sentiment of a place cannot be linked with aggregate health outcomes. This study used a comparably smaller geography to make its analysis compared to other Twitter-based studies [2,25,39], which resulted in fewer geographic observations and fewer tweets per observation, which limits the generalizability of this study. Nonetheless, this study demonstrates that how these measures are constructed must be addressed to ensure their validity. More care needs to be taken to understand the underlying racial/ethnic and class formations that uniquely shape sentiment and language. The existing measures do not adequately account for the unique ways different racial/ethnic groups and social classes express emotions. Future work in this area should do more specific coding by race, conducting quality assessment checks with specific racial and ethnic groups. Further, to understand the full scope of how Twitter sentiment matters for local health, more should be done to unpack the intervening factors that turn emotions into health outcomes. One can be happy, for example, but still partake in poor health behaviors that lead to poor health. How do forces like efficacy, the drive one has to involve themselves in proactive health habits, work with emotions to lead to health outcomes?

In closing, sentiment expressed through social media sources offer health care professionals and policymakers exciting new ways to determine health and well-being within and across cities. Highly nuanced data, however, requires highly nuanced preparation.
